# Supplementary material for: Bacterial coinfection restrains antiviral CD8 T-cell response via LPS-induced inhibitory NK cells
Source: Nat Commun. 2018 Oct 8;9:4117. doi: 10.1038/s41467-018-06609-z (PMC6175863; doi:10.1038/s41467-018-06609-z)
Supplement: Supplementary file 1 — Supplementary Information [file 41467_2018_6609_MOESM1_ESM.pdf]

Supplementary Information

Bacterial coinfection restrains antiviral CD8 T cell response via LPS-induced inhibitory NK cells

Tobias Straub, Marina S. Freudenberg, Ulrike Schleicher, Christian Bogdan, Georg Gasteiger and Hanspeter Pircher

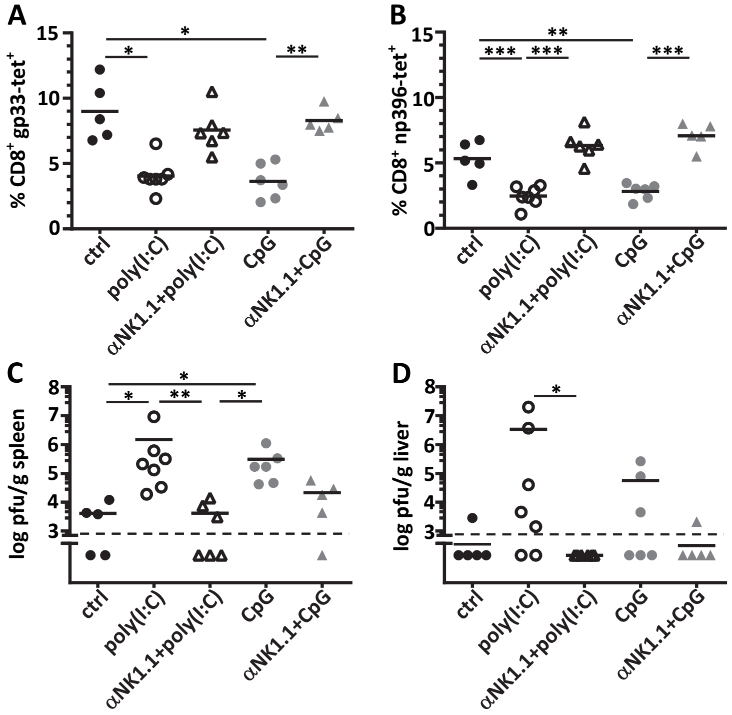

Supplementary Figure 1. Poly(I:C) and CpG oligodeoxynucleotides (ODN) treatment leads to NK cell-mediated impairment of the antiviral CTL response in LCMV-infected mice

NK cell-depleted and non-depleted B6 mice were infected with 200 pfu LCMV-WE. As indicated, they additionally received 50 µg poly(I:C), 50 µg CpG ODN or PBS as control (ctrl) at day 1 p.i.. Mice were analyzed at day 8 after LCMV infection. (A, B) gp33- and np396-tetramer^{+} (tet) CD8 T cells in percent of spleen cells. (C, D) Viral titers in spleen and liver. Data of individual mice from 2 independent experiments (n = 5-7) with 2-3 mice per group are shown; horizontal bars represent the mean values. Dashed lines indicate detection limits. * $p < 0.05$, ** $p < 0.01$, *** $p < 0.001$; one-way ANOVA with Turkey-Kramer post-test (B) or Kruskal-Wallis Test with Dunn’s post-test (A, C, D).

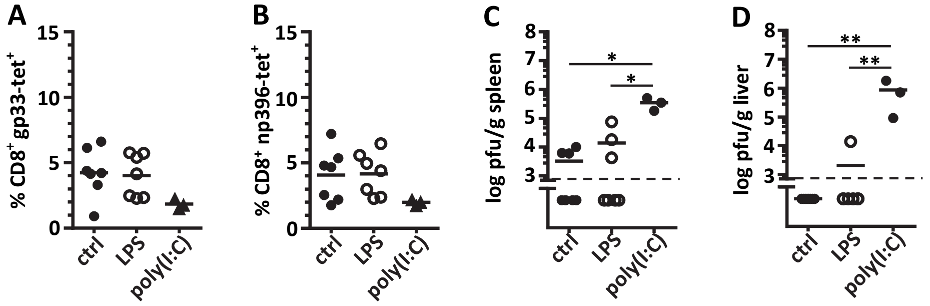

Supplementary Figure 2. Poly(I:C) but not LPS-treatment suppresses the anti-LCMV immune response in TLR2/4-deficient mice

TLR2/4-deficient mice were infected with 200 pfu LCMV-WE. One day later, they were treated with 1 µg LPS, 50 µg poly(I:C) or PBS (ctrl). Mice were analyzed at day 8 p.i.. (A, B) gp33- and np396-tetramer$^+$ (tet) CD8 T cells in percent of spleen cells. (C, D) Viral titers in spleen and liver. Data of individual mice from one (poly(I:C)) or three (ctrl, LPS) independent experiments (n = 4-7) with 2-3 mice per group are shown; horizontal bars represent the mean values. Dashed lines indicate detection limits. * $p < 0.05$, ** $p < 0.01$; Kruskal-Wallis Test with Dunn’s post-test.

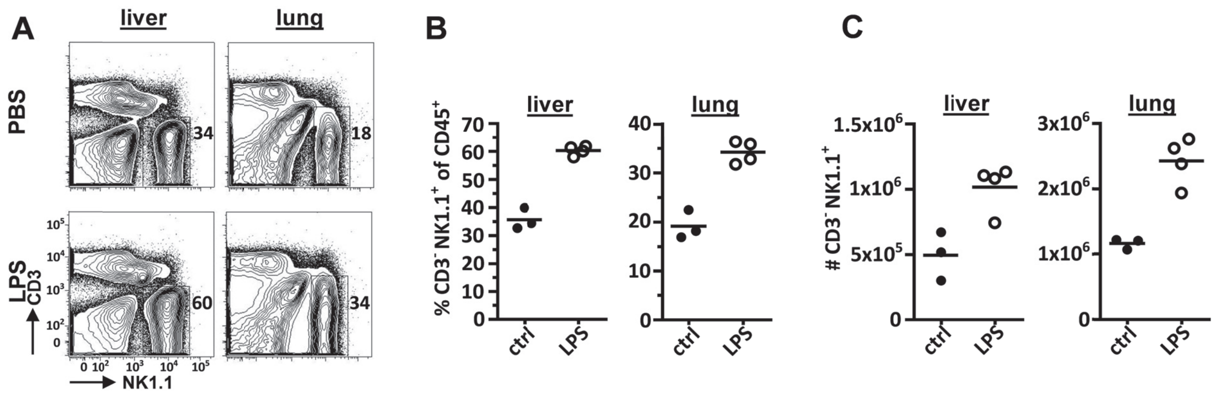

Supplementary Figure. 3. LPS-treatment leads to increased NK cell numbers in liver and lungs of LCMV infected mice

B6 mice were infected with LCMV and treated with LPS (1 µg) or PBS (ctrl) one day later. At day 4 p.i. NK cell ($CD3^-NK1.1^+$) frequencies in livers and lungs were determined. (A) Representative contour plots are shown. Numbers indicate percentage of NK ($CD3^-NK1.1^+$) cells. (B, C) Summarizing scatter plots showing percentage of NK cells among $CD45^+$ cells and absolute numbers (#) of NK cells. Data of individual mice from one experiment with 3-4 mice per group are shown; horizontal bars represent the mean values.

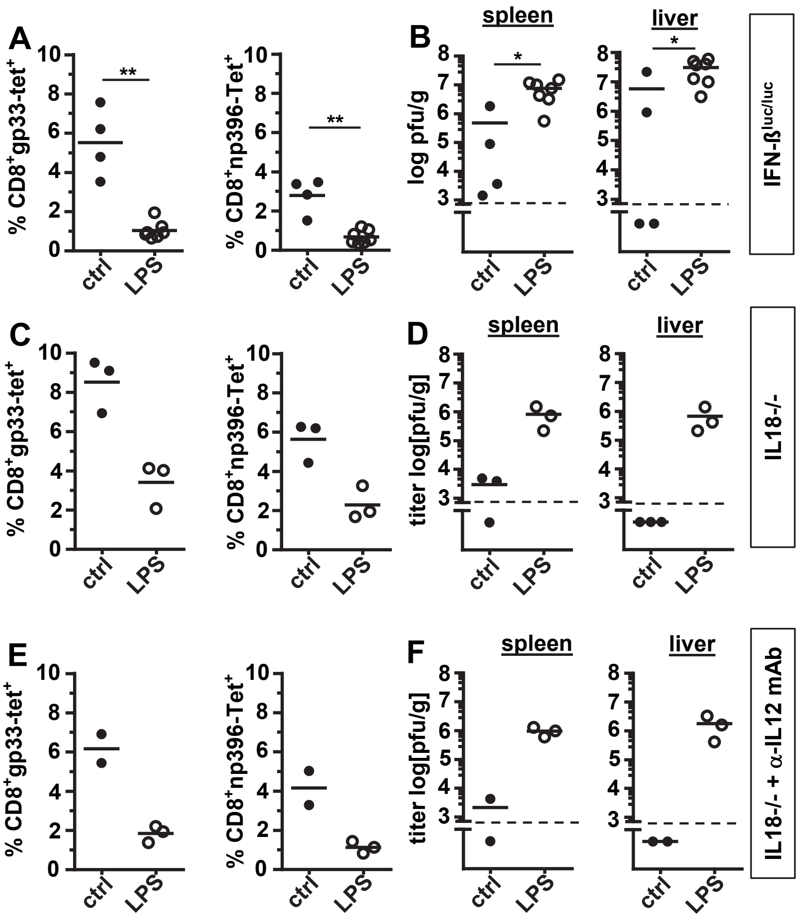

Supplementary Figure. 4. IFN-ß, IL-12 and IL-18 are dispensable for negative T cell regulation by LPS-activated NK cells

IFN-ß$^{luc/luc}$ (A, B), IL-18-deficient (C, D) and anti-IL-12 mAb-treated IL-18-deficient (E, F) mice were infected with LCMV and one day later received LPS (1 µg) or PBS as control. At day 8 p.i. mice were sacrificed. (A, C, E) Frequency of $CD8^+$gp33-tet^+ and $CD8^+$np396-tet^+ cells in spleens; (B, D, F) viral titers in spleen and liver. Data of individual mice from (A, B) 3 independent experiments (n = 4-7) with 1-3 mice per group, and (C-F) one experiment with 2-3 mice per group are shown; horizontal bars represent the mean values. Dashed lines indicate detection limits. * $p < 0.05$, ** $p < 0.01$; Mann-Whitney Test.

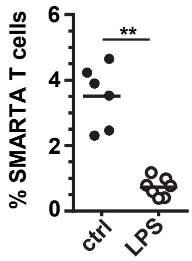

Supplementary Figure 5. LPS inhibits clonal expansion of LCMV-specific SMARTA CD4 T cells

10^4 LCMV-specific SMARTA CD4 T cells were transferred into B6 mice, which were subsequently infected with LCMV. One day later, mice were treated with LPS (1 µg) or PBS as control (ctrl). At day 7 p.i. frequencies of SMARTA CD4 T cells in the spleen of recipient mice were determined. Data of individual mice from two independent experiments (n = 6-7) with 3-4 mice per group are shown; horizontal bars represent the mean values. ** $p < 0.01$; Mann-Whitney Test.

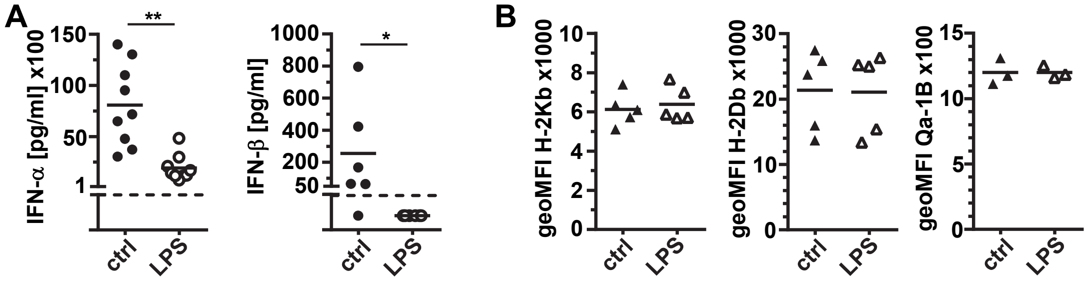

Supplementary Figure 6. Decreased levels of type I IFN in sera of LPS-treated LCMV-infected mice

(A) B6 mice were infected with LCMV and treated one day later with LPS or PBS as control (ctrl). 24 hours after LPS-treatment mice were bled and the concentrations of IFN-α (n = 9) and IFN-β (n = 6) in the sera were determined. (B) P14 T cells were transferred into NK cell-depleted B6 recipient mice, which were subsequently infected with LCMV. After one day, they received LPS or PBS as a control (ctrl). At day 5 p.i. transferred P14 T cells were analyzed for the expression of the indicated MHC class I (like) molecules (n = 5). Data of individual mice from 2-4 independent experiments with 2-3 mice per group are shown; horizontal bars represent the mean values, dashed lined indicate the detection limit. * $p < 0.05$, ** $p < 0.01$; unpaired t-Test with Welch correction (IFN-α) or Mann-Whitney Test (IFN-ß).

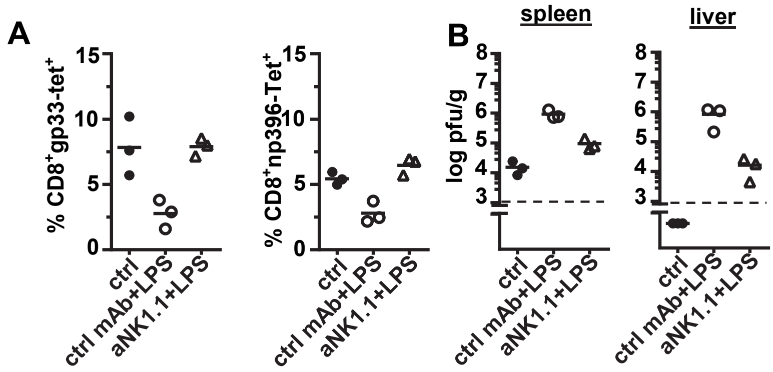

Supplementary Figure 7. Anti-NK1.1 but not isotype control mAb prevent the negative effect of LPS on anti-LCMV immunity

B6 mice were treated at day 2 and day 1 before LCMV infection with anti-NK1.1 mAb or isotype control mAb or left untreated. One day after infection, mice received LPS (1 µg) or PBS as control (ctrl). At day 8 p.i. mice were sacrificed. (A) Frequency of $CD8^{+}$gp33-tet^{+} and $CD8^{+}$np396-tet^{+} cells in spleens; (B) viral titers in spleen and liver. Data of individual mice from one experiment with 3 mice per group are shown; horizontal bars represent the mean values. Dashed lines indicate detection limits.

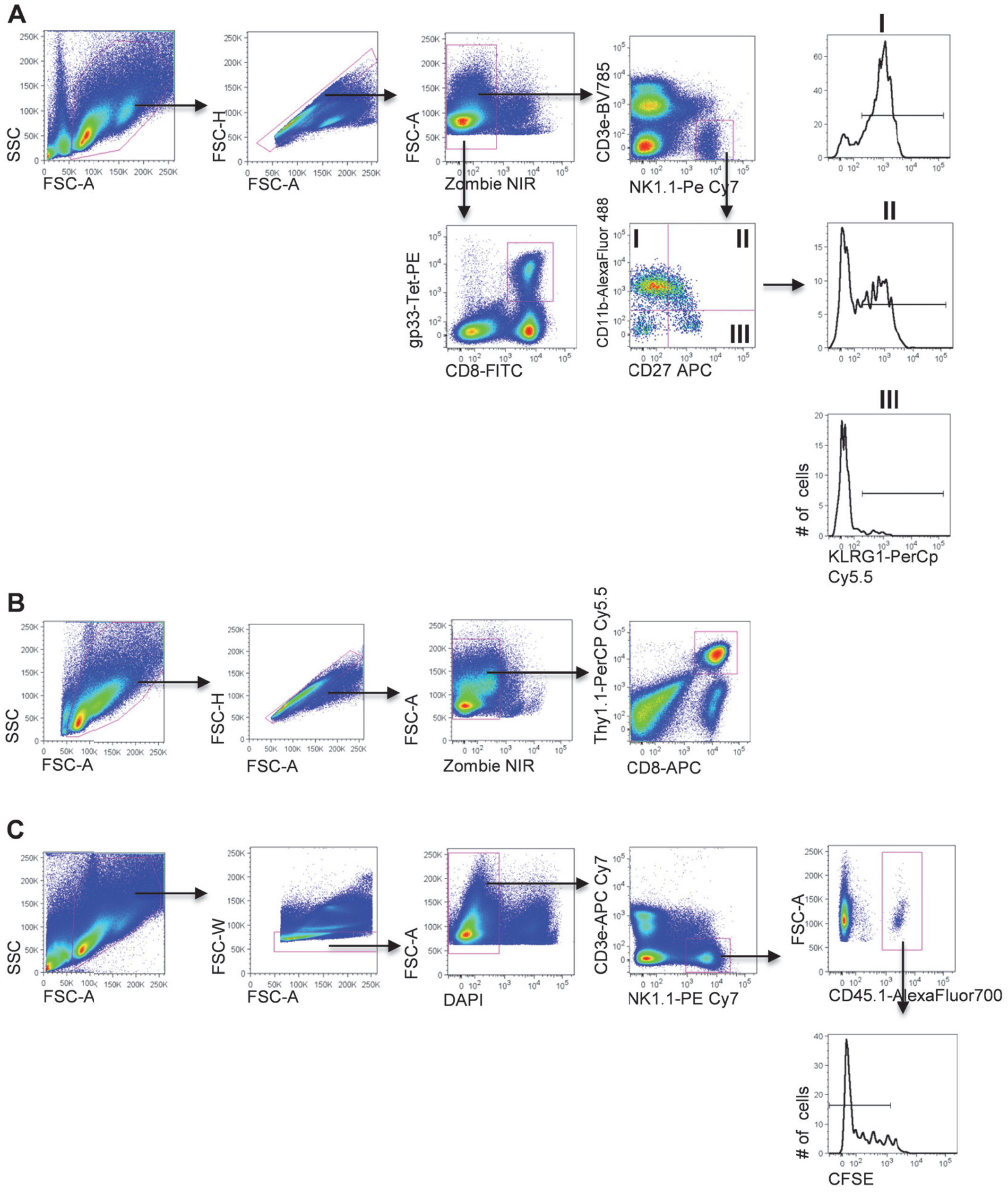

Supplementary Figure 8. Gating strategies

(A) Gating strategy used to analyze the data shown in Figure 1, Figure 2, Figure 4 A and C, Figure 5, Figure 6, Supplementary Fig. 1, Supplementary Fig. 2, Supplementary Fig. 3, Supplementary Figure 4, and Supplementary Fig. 6.

(B) Gating strategy used to analyze the data shown in Figure 3.

(C) Gating strategy used to analyze the data shown in Figure 4 D.
